# Volatile organic compounds shape belowground plant–fungi interactions

**DOI:** 10.3389/fpls.2022.1046685

**Published:** 2022-12-06

**Authors:** Nguyen Hong Duc, Ha T. N. Vo, Cong van Doan, Kamirán Áron Hamow, Khac Hoang Le, Katalin Posta

**Affiliations:** ^1^ Institute of Genetics and Biotechnology, Department of Microbiology and Applied Biotechnology, Hungarian University of Agriculture and Life Sciences (MATE), Godollo, Hungary; ^2^ Plant Disease Laboratory, Department of Plant Protection, Faculty of Agronomy, Nong Lam University, Ho Chi Minh, Vietnam; ^3^ Molecular Interaction Ecology, German Centre for Integrative Biodiversity Research (iDIV), Leipzig, Germany; ^4^ Agricultural Institute, Centre for Agricultural Research, Martonvásár, Hungary

**Keywords:** belowground volatile organic compounds, arbuscular mycorrhizal symbiosis, ectomycorrhiza, pathogenic fungi, belowground plant-fungi interactions, belowground VOC research methods

## Abstract

Volatile organic compounds (VOCs), a bouquet of chemical compounds released by all life forms, play essential roles in trophic interactions. VOCs can facilitate a large number of interactions with different organisms belowground. VOCs-regulated plant-plant or plant-insect interaction both below and aboveground has been reported extensively. Nevertheless, there is little information about the role of VOCs derived from soilborne pathogenic fungi and beneficial fungi, particularly mycorrhizae, in influencing plant performance. In this review, we show how plant VOCs regulate plant-soilborne pathogenic fungi and beneficial fungi (mycorrhizae) interactions. How fungal VOCs mediate plant–soilborne pathogenic and beneficial fungi interactions are presented and the most common methods to collect and analyze belowground volatiles are evaluated. Furthermore, we suggest a promising method for future research on belowground VOCs.

## Introduction

Volatile organic compounds (VOCs) are a mixture of low molecular-weight compounds originating from different types of organisms (Maffei et al., 2011). Under biotic (insects, beneficial fungi, pathogenic fungi, bacteria) and abiotic (heat, drought, UV radiation) stresses, plants often release complex VOC bouquets. Plant VOCs are essential in communication between plants and other organisms (Dudareva et al., 2006), which has been demonstrated in the laboratory and in agricultural systems (Kessler and Baldwin, 2001; Baldwin et al., 2002; Turlings and Erb, 2018). In previous research, volatiles emitted from microorganisms such as bacteria and fungi have been investigated less than VOCs emitted from plants (Effmert et al., 2012; Junker and Tholl, 2013; Weisskopf, 2013; Penuelas et al., 2014).

Microbial VOCs are released by microorganisms such as bacteria and beneficial and pathogenic fungi (Korpi et al., 2009; Thorn and Greenman, 2012). Volatile organic compound profiles can be substantially altered by pathogen-derived VOCs, and can therefore function as biomarkers for detection, differentiation, and characterization or even forecast of early infections (Li et al., 2019; Hamow et al., 2021). More than 100 bacteria and fungi produce soil microbial VOCs (Effmert et al., 2012), and approximately 250 fungal VOCs have been described (Morath et al., 2012; Roze et al., 2012). Plants can perceive microbial VOCs from a distance and prime plant responses to microorganisms (Bailly and Weisskopf, 2012; Effmert et al., 2012; Bitas et al., 2013; Schmidt et al., 2015). Microbial VOCs can potentially mediate plant–microbe interactions (Moisan et al., 2020a; Moisan et al., 2020b; Xu et al., 2021). Microbial VOCs can diffuse through the soil environment and potentially affect plant growth and defense (Piechulla et al., 2017; Tyagi et al., 2018). Bacterial VOCs can increase plant growth and trigger systemic resistance and also influence motility and antibiotic resistance in other bacteria (Ryu et al., 2003; Ryu et al., 2004; Lee et al., 2012; D’Alessandro et al., 2014; Park et al., 2015). Similarly, VOCs emitted by pathogenic and beneficial microorganisms can promote plant growth (Velásquez et al., 2020b), and microbial volatiles can improve plant tolerance and sustain plant growth (Liu and Zhang, 2015; Jalali et al., 2017; Camarena-Pozos et al., 2019).

Volatile organic compounds can facilitate many interactions between below- and aboveground organisms (Schulz and Dickschat, 2007; Das et al., 2012; Junker and Tholl, 2013). Compared with aboveground VOCs, belowground VOCs are challenging to evaluate because of the nonhomogeneous soil environment. The difficulty results in technical limitations in collecting volatiles (Tholl et al., 2021). Since the first investigation of Baldwin and Schultz (1983), VOC-regulated plant–plant or plant–insect interactions both below and aboveground have been investigated extensively (Bruce et al., 2005; Baldwin et al., 2006; Kegge and Pierik, 2010; Clavijo McCormick et al., 2012; Effah et al., 2019; Effah et al., 2022). However, much less known about roles of VOCs originating from soilborne pathogenic and beneficial fungi, particularly mycorrhizae, in affecting plant performance. In addition, how exposure to fungal VOCs affects plant resistance or tolerance to aboveground and belowground herbivory has not been addressed. This review shows (1) how plant VOCs mediate plant–soilborne pathogenic and beneficial fungi (mycorrhizae) interactions; (2) how fungal VOCs modulate plant–soilborne pathogenic and beneficial fungi interactions and (3) we describe the most common methods to collect and analyze belowground volatiles and a promising method for future research on belowground VOCs is introduced.

## Volatile organic compounds in plant–pathogenic fungi interactions

### Plant belowground volatile organic compounds and effects on fungal pathogens

**Figure 1:**
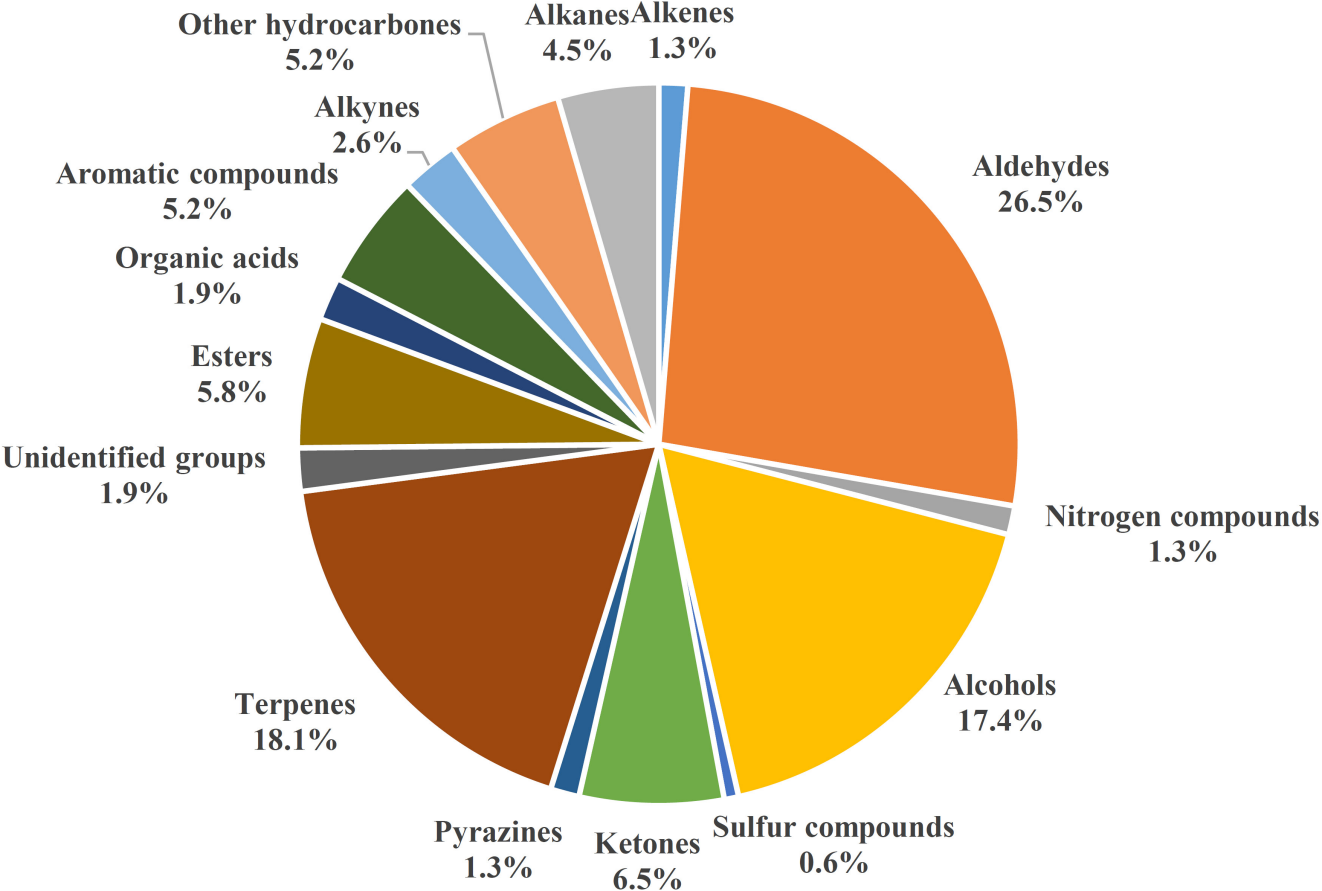
Diversity of plant root VOCs, 155 volatile compounds from different plants collected from 2016 to 2022.

Because of negative effects of chemical use in plant protection, analyzing production patterns of VOCs in root tissues is increasingly important because of potential VOC roles in belowground biotic interactions, particularly those with fungal pathogens. Numbers of root VOCs that have been identified and investigated have increased in recent years. In 2015, relatively few root volatiles (39 compounds) were known in maize, barley, bean (*Vicia faba*), and *Arabidopsis thaliana* (reviewed by Schenkel et al., 2015). However, hundreds more volatile compounds emitted by roots of diverse plant species have since been reported (Cordovez et al., 2017; Schenkel et al., 2018; Moisan et al., 2019). With the model plant *A. thaliana*, the focus has been on different functions of root volatiles in invasive and noninvasive conditions (Casarrubia et al., 2016; Cordovez et al., 2017; Schenkel et al., 2018; Moisan et al., 2019). Volatile organic compounds of many other plants have also been investigated. Volatile organic compounds in Solanaceae, including pepper (*Capsicum annuum)* (Kihika et al., 2017) and tomato (Kihika et al., 2017; Kirwa et al., 2018); Brassicaceae, including *Brassica rapa* (Moisan et al., 2020); and the cucurbit family, including cucumbers *Cucumis metuliferus* CM3 and line Xintaimici in relation to *Meloidogyne incognita* (Xie et al., 2022), as well as those in non-cultivated plants, including spotted knapweed *Centaurea stoebe* (Gfeller et al., 2019), have been scanned and investigated for antifungal activity or ability to increase plant defense against pathogens and herbivores. These root VOCs have been grouped into 15 biosynthetic origins/chemical classes in **Figure 1** (**Table S1**). Because of limited pesticide use for the control of fungal pathogens, some antifungal VOCs may be promising control agents. However, antimicrobial activity of VOCs can vary with origin, dose, and application form, and possible phytotoxic effects and effects on human health of antifungal VOCs need to be investigated in order to develop effective and safe biocontrol strategies (Kaddes et al., 2019a).

Volatile organic compounds are classified into different chemical groups depending on plant species, genotype, sex, development stage (**Table 1** and **Table S1**) (Schenkel et al., 2015; Delory et al., 2016a; Delory et al., 2016b; Kihika et al., 2017; Kindlovits et al., 2018; Murungi et al., 2018; Xie et al., 2022). One of the most common groups is terpenoids, which include the sesquiterpenes (E)‐β‐caryophyllene, daucadiene, (E)‐α‐bergamotene, humulene, (E)‐β‐farnesene, and three putative petasitene isomers (petasitene 1–3) and the monoterpenes α‐pinene and β‐myrcene (Gfeller et al., 2019; Gulati et al., 2020). In *Achillea collina*, active volatile constituents included alismol, (E)-β-farnesene, β-sesquiphellandrene and neryl esters, heptadecen-7-one, albene and β-pinene, linoleic acid, 2,4,6-decatrienoic acid piperideide, sterols, and some triterpenes (Kindlovits et al., 2018). Other major groups of root volatiles include aldehydes, alcohols, n-alkanes, and ketones. Following strong mechanical injury in barley plants at each developmental stage, the four main volatile aldehydes were characterized and included hexanal, (E)-hex-2-enal, (E)-non-2-enal, and (E,Z)-nona-2,6-dienal (Delory et al., 2016a). The volatile organic compounds released by roots vary depending on the biotic stress agent that is causing damage to the plant. Tomato roots infected by *Fusarium oxysporum* emit VOCs such as benzonitrile, benzothiazol, dimethyl trisulfide, and formic acid, which have antifungal activities, and a terpene-like compound, which activates antagonistic response; whereas healthy tomato plants release n-alkanes, beclomethasone dipropionate, p-cymene, decanal, and 3-carene, which are compounds without antimicrobial activity or special role (Gulati et al., 2020). Belowground VOCs can affect root-associated microbes, including belowground fungal pathogens. Antimicrobial VOCs biosynthesized in natural hosts are typically at low levels, but the substantial antagonistic activity is promising. Contact-independent antagonisms by VOCs indicate potential for a single application with uniform exposure. Such exposure decreases the likelihood of unaffected host microbial refugia being re-colonized by pathogens after dissipation or degradation of an inhibitory compound (Gabriel et al., 2018). Black pepper-associated bacterium *Pseudomonas putida* BP25 was isolated from a root endosphere, and endophytic colonization by PpBP25 protected black pepper, ginger, and *Arabidopsis* against *Phytophthora capsici*, *Pythium myriotylum*, *Giberella moniliformis*, *Rhizoctonia solani*, *Athelia rolfsii*, *Colletotrichum gloeosporioides*, and the plant parasitic nematode *Radopholus similis* because of the release of volatile substances such as pyrazine derivatives (Sheoran et al., 2015). Pyrazine derivates such as 2,5-dimethyl pyrazine, 2-methyl pyrazine, dimethyl trisulfide, 2-ethyl 5-methyl pyrazine, and 2-ethyl 3,6-dimethyl pyrazine have *in vitro* inhibitory activity against the oomycete pathogens *Ph. capsici* and *P. myriotylum*; the fungal pathogens *R. solani*, *C. gloeosporioides*, *A. rolfsii*, *G. moniliformis*, and *Magnaporthe oryzae*; the bacterial pathogen *Ralstonia pseudosolanacearum*; and the plant parasitic nematode *Radopholus similis*. (Kihika et al., 2017; Murungi et al., 2018) In addition to pyrazine derivatives, the VOC dimethyltrisulfide of BP25 exhibits soil fumigant activity against *Ph. capsici*; *R. solani*, *A. rolfsii*, *C. gloeosporioides*, and *G. moniliformis*; and *R. similis* (Agisha et al., 2019). Thus, *P. putida* BP25 and its VOCs have promise as applications for eco-friendly disease management in sustainable agriculture. *Botrytis cinerea*, a necrotrophic fungus with a wide range of hosts, is extremely sensitive to monoterpenes, such as (+)-limonene, in *in vitro* applications (Simas et al., 2017), which inhibit fungal mycelial growth and spore germination. The eight-carbon oxylipin 1-octen-3-ol is the primary factor suppressing conidia germination and mycelial growth of *Aspergillus nidulans* (Herrero-Garcia et al., 2011).

**Table 1:** Plant root VOCs and its properties.

Plant	VOC compounds	Properties	References
*Carex arenaria*	γ-capro; γ-deca; γ-nonalactone	attract benefit bacteria from bulk soil	Schulz-Bohm et al., 2017
*Cucumis metuliferus* CM3	Creosol	attract and kill *M. incognita*	Xie et al., 2022
Poplar	salicylaldehyde	play a role as a nematicide	Lackus et al., 2018
*Cucumis metuliferus* CM3	Benzene, (methoxymethyl)	repel *M. incognita*	Xie et al., 2022
Pepper	Thymol	repel root-knot, cyst, and stubby root nematodes	Kihika et al., 2017
*Centaurea stoebe*; tomato	(E)‐β‐caryophyllene; daucadiene; (E)‐α‐bergamotene; humulene; (E)‐β‐farnesene; petasitene 1–3; β‐myrcene	effect on the germination and growth of different sympatric neighbors	Gfeller et al., 2019; Gulati et al., 2020
*Centaurea stoebe*; tomato; spinach; pepper; poplar	α‐pinene		Kihika et al., 2017; Murungi et al., 2018; Lackus et al., 2018; Gfeller et al., 2019; Gulati et al., 2020
Cucumber line Xintaimici, Tomato, spinach; pepper	Tridecane	attract second-stage larvae (J2) of *M. incognita;* Simas et al., 2017	Kihika et al., 2017; Murungi et al., 2018; Xie et al., 2022
Tomato; Pepper	p-cymene		Kihika et al., 2017; Gulati et al., 2020
Tomato	Sabinene		Murungi et al., 2018
Tomato, spinach; pepper	Limonene; 2-(methoxy)-3-(1 methylpropyl)pyrazine		Kihika et al., 2017; Murungi et al., 2018
Tomato, spinach	2-isopropyl-3-methoxypyrazine		Murungi et al., 2018
*Cucumis metuliferus* CM3	2-Penten-1-ol, (Z)-		Xie et al., 2022
Cucumber line Xintaimici; pepper; tomato	Methyl salicylate		Kihika et al., 2017; Murungi et al., 2018; Xie et al., 2022
*Cucumis metuliferus* CM3	1-Nonyne	improvement plant resistance to *M. incognita*	Xie et al., 2022
*Carex arenaria*	Benzonitrile		Schulz-Bohm et al., 2017
*Carex arenaria*	Benzofuran		Schulz-Bohm et al., 2017
Not given	Limonene	inhibited the fungal mycelial growth and spore germination of *Botrytis cinerea*	Simas et al., 2017
Barley	methy pro-2-enoate and methyl propanoate	suppressed the mycelial growth and prohibited spore germination of *Fusarium culmorum* and *C. sativus*	Kaddes et al., 2019b
Tomato	benzonitrile, benzothiazol, dimethyl trisulfide	Antifungal activity to *Fusarium oxysporum*	Gulati et al., 2020

Two organic esters (methyl pro-2-enoate and methyl propanoate) suppress mycelial growth of the fungi *Fusarium culmorum* and *Cochliobolus sativus* when in direct contact, whereas with indirect contact, the VOCs cause a decrease in the outflow of K^+^ ions into the intracellular medium and an increase in the permeability of pathogenic spore membranes (Kaddes et al., 2019b). Because activity of proton pumps must guarantee the efflux of H^+^ ions into the intracellular medium to retain electrical charges on either side of the membrane at equilibration in order to adjust for K^+^ imbalance, dramatic changes can occur in the pH of the intracellular medium and prohibit spore germination (Kaddes et al., 2019b). Therefore, roles of root volatiles in regulating belowground microbiomes *via* effects on microbial communities and attraction of beneficial microbial species have been the focus of research. Despite the bright prospects to control fungal diseases, applications of belowground volatiles in sustainable agriculture need to be investigated further (Sharifi et al., 2022).

### Effects of volatile organic compounds produced by fungal pathogens on plants

Pathogenic fungi obtain nutrients by either feeding on living host plant cells (biotrophic pathogens) or killing cells (necrotrophic pathogens). Notably, volatile compounds emitted by pathogenic fungi are different from those emitted by plant roots (Schenkel et al., 2015; Gulati et al., 2020). Among pathogen genera, fungal volatile compounds have been characterized for many species (Fiers et al., 2013; Casarrubia et al., 2016; Werner et al., 2016; Cordovez et al., 2017; Cordovez et al., 2018; Martín-Sánchez et al., 2020; Moisan et al., 2020). Pathogen-produced VOCs have low chemical diversity and are most likely used as info-chemicals or chemical stimuli to attract or repel interacting organisms (Gulati et al., 2020). Pathogenic fungi and the volatiles emitted by such fungi negatively affect plant growth. Fungal volatile compounds such as 1-octen-3-ol, 2-phenylethanol, 3-methyl-1-butanol, 1-hexanol, 3-octanol, 3-octanone, and trans-2-octenal (**Table 2**) are classified as phytotoxic (Werner et al., 2016). The compound 1-octen-3-ol represses root growth and causes cotyledon bleaching of *A. thaliana* seedlings at low concentrations *via* H_2_O_2_ production (Splivallo et al., 2007) and impairs seed germination (Lee et al., 2014). Volatiles emitted by the belowground fungal pathogens *Serratia plymuthica* and *F. culmorum* affect root and shoot growth of maize by limiting the availability of micronutrients such as Fe, Zn, Cu, and Mo (**Table 2**) in roots (Martín-Sánchez et al., 2020). The fungus *F. acuminatum* releases volatiles into soil that prohibit growth and decrease shoot length and root surface area and biomass in tomato (Gulati et al., 2020). The fungi *F. culmorum* and *Cochliobolus sativus* produce VOCs that decrease leaf surface area and mean root length in barley (Fiers et al., 2013). Volatiles emitted by the pathogenic fungi *R. solani* and *F. oxysporum* f. sp. *raphani* decrease the root growth rate of *B. rapa* seedlings (Moisan et al., 2021).

**Table 2:** Fungal VOCs and its effect to plant host.

Plant host/fungi	VOC compounds	Properties	References
Maize*/ Serratia plymuthica; Fusarium culmorum*	Not given	Iimited the availability of micronutrients such as Fe, Zn, Cu, and Mo in the root	Martín-Sánchez et al., 2020
Tomato*/ Fusarium oxysporum*	branched alcane, dodecane, eicosane, docosane, naphthalene, beclomethasone dipropionate	Prohibited plant growth and curtailed shoot length and root parameters, as well as lessened root surface and biomass	Gulati et al., 2020
*Brassica rapa/ R. solani*, *Fusarium oxysporum* f.sp*. raphani*	3-octanol, 3-octanone	Diminished the root growth rate of *Brassica rapa* seedlings	Moisan et al., 2021
*A. thaliana/ R. solani*	1-octen-3-ol, 2-phenylethanol, 3-methyl-1-butanol, 1- hexanol, 3-octanol, 3-octanone, trans-2-octenal	Inhibited plant growth	Werner et al., 2016; Cordovez et al., 2017
*A. thaliana/ R. solani*	Unidentified	Plant growth promoted by altering root architecture and enhancing root biomass; reduced aboveground resistance to the herbivore *Mamestra brassicae*	Cordovez et al., 2017
*Brassica rapa/ R. solani*, *Fusarium oxysporum, Ulocladium atrum* and *Phoma leveillei*	Not given	Stimulated root and plant growth, flowering, accelerating plant bolting, bud and flower production, improved reproductive success; enhanced plant resistant to cabbage root fly *Delia radicum* and large cabbage white butterfly *Pieris brassicae*	Moisan et al., 2020a
*Brassica rapa / F. oxysporum*	Not given	Inhibited root-knot nematode *M. incognita* egg hatching and development of cyst nematode *Heterodera schachtii*	Terra et al., 2018; Moisan et al., 2021
Arabidopsis/ *Penicillium aurantiogriseum*	Not given	modify root metabolism and architecture, and improve nutrient and water use efficiencies	García-Gómez et al., 2020
*-/ Fusarium culmorum*	α-Terpinene, β-Phellandrene, 3-Carene, and Camphene	Reduced swimming and swarming motility bacteria, *Collimonas pratensis* Ter291 and *Serratia plymuthica* PRI-2C	Schmidt et al., 2016
*Tricholoma vaccinum* (EM fungi)	Produced monoterpene limonene, sesquiterpene β-barbatene	Antimicrobial activity	Abdulsalam et al., 2021
*Tilia americana/Tuber borchii* (EM fungi)	Produced 29 volatiles including alcohols, aldehydes, and ketones	These VOCs may facilitate ectomycorrhizal fungi establishment	Menotta et al., 2004
*Populus/Laccaria bicolor* (EM fungi)	Released sequiterpene thujopsene	Increased Populus lateral root formation and root hair length in the pre-symbiotic phase, facilitating ectomycorrhizal fungi establishment	Ditengou et al., 2015
*Tricholoma vaccinum* (EM fungi)	Emitted geosmin	Improved sporulation and spore germination in AMF. This volatile may also be important in ectomycorrhizal fungi establishment	Abdulsalam et al., 2021
*Rhizophagus irregulari* (AMF)	Produced unknown volatiles	Directly suppressed growth and extension of fungal pathogens, *F. oxysporum, F. graminearum, Verticillium dahlia, Rhizoctonia solani*	Zhang et al., 2018
*Gigaspora margarita* (AMF)	Emitted unknown volaties	Increased density and number of lateral roots of A. thaliana (non-host plant for AMF) and *Lotus japonicus*	Sun et al., 2015
-/ AM genus Glomus	Not given	Improved biotic stress tolerance in an array of plants attacked by herbivores	Dowarah et al., 2021
*Medicago truncatula/Rhizophagus irregularis*	Specifically released limonene	This volatile may help plant recognize the symbiotic mycorrhizal fungi	Dreher et al., 2019
Tomato /*R. irregularis*	Increased methyl salicylate	Attracted the aphid parasitoid *Aphidius ervi*	Volpe et al., 2018
*Asclepias curassavica* /*Funneliformis mosseae*	Increased 3-hexenyl acetate, hexyl acetate, methyl salicylate	modified plant attractiveness to insect behavior	Meier and Hunter, 2019
Grapevine/*F. mosseae*	Increased benzaldehyde, geraniol, 2–hexenal, 3–hexenal	Improved plant defenses against pathogen/herbivore attack Improved plant defences against pathogen/herbivore attack	Velásquez et al., 2020b
*Elymus nutans/ F. mosseae*	Increased D-Limonene, p-Xylene, 1,3-Diethylbenzene		Zhang et al., 2022
Grapevine*/ F.. mosseae*	C13–norisoprenoid *β*–ionone decline	Improved plant resistance to water stress	Ju et al., 2018
*Medicago sativa /Rhizophagus irregularis*	Volatization of inorganic Asenic	Decreased As toxicity in the host plant	Li et al., 2021

AMF, arbuscular mycorrhizal fungi; EM fungi, ectomycorrhizal fungi.

In addition to negative effects on plants, many pathogenic fungal VOCs are growth manipulators, because the VOCs affect plant architecture and increase growth (Werner et al., 2016). Many of those VOCs are alcohols, pyrones, phenols, sesquiterpenes, ketones, and aldehydes, which can affect plant growth and architecture (Casarrubia et al., 2016; Cordovez et al., 2018; Fincheira and Quiroz, 2018; Moisan et al., 2019). Volatile organic compounds emitted by the fungal root pathogen *R. solani* promote *in vitro* growth of early developmental stages of *A. thaliana* by altering root architecture and increasing root biomass (Cordovez et al., 2017) and increase root growth of *B. rapa* (Moisan et al., 2020a). Increases in growth can benefit pathogens by enlarging the habitat for pathogenic colonization of surfaces and survival (Cordovez et al., 2017; Moisan et al., 2019; Moisan et al., 2020). Fungal volatiles emitted by *R. solani* and *Phoma leveillei* stimulate not only plant growth but also flowering by accelerating plant bolting and bud and flower production, which improves reproductive success (Moisan et al., 2019). The ability of fungal pathogens to modulate plant growth *via* VOCs is likely widespread, because VOCs act as an “alert” signal to plants, which accelerate growth by upregulating genes involved in auxin or cytokinin signaling while downregulating genes involved in ethylene or jasmonic acid signaling (Sanchez-Lopez et al., 2016; Cordovez et al., 2017; Martínez-Medina et al., 2017b; Li and Kang, 2018).

Fungal VOCs increase plant protection by inducing host defense systems and resistance against pathogens *via* different mechanisms (Werner et al., 2016). One important mechanism is to change the balance of K^+^ ions flow and disturb the pH gradient, which inhibits fungal mycelial growth and spore germination (Kaddes et al., 2019b). Naphthalene and monoterpenes (p-cymene, 3-carene) produced in tomato roots in response to *F. oxysporum* have antibacterial effects (Gulati et al., 2020). Plant VOC emission profiles can also change after infection with a fungal pathogen, leading to chemical protection in plants and preventing further fungal pathogen colonization or attracting specific beneficial microorganisms with antifungal properties (Schulz-Bohm et al., 2017; Gulati et al., 2020).

Notably, soilborne pathogenic fungi can improve plant resistance to above- and belowground herbivory. Resistance to the cabbage root fly *Delia radicum* (Diptera: Anthomyiidae) and the large cabbage white butterfly *Pieris brassicae* (Lepidoptera: Pieridae) increase with exposure to volatiles of the pathogenic fungi *R. solani*, *F. oxysporum*, *Ulocladium atrum*, *and P. leveillei* (Moisan et al., 2019). Fungal VOCs also negatively affected *D. radicum* development rate and *P. brassicae* caterpillar performance (Cordovez et al., 2017; Moisan et al., 2019; Moisan et al., 2020b). Exposure of roots to fungal VOCs can alter primary and lateral root architecture, which leads to changes in plant chemistry and morphological characteristics and negatively affects the performance of root herbivores by delaying insect growth and accumulation of body mass (Ditengou et al., 2015; Casarrubia et al., 2016). Fungal VOCs can also promote the accumulation of glucosinolates in leaves or main roots, which diminishes leaf caterpillar performance or slows larval development (Aziz et al., 2016). In addition, volatiles produced by soilborne fungi can affect nematode development and behavior. Volatiles emitted by some *F. oxysporum* strains inhibit egg hatch in the root-knot nematode *Meloidogyne incognita* and slow development of the cyst nematode *Heterodera schachtii* (Terra et al., 2018; Moisan et al., 2021). Thus, volatiles from soilborne fungi not only negatively affect or modulate plant growth but also diffuse through the soil matrix to help plants attract disease antagonists or natural enemies for defense.

## Volatile organic compounds in mycorrhizal symbiosis

### Volatile organic compounds during mycorrhizal establishment

Arbuscular mycorrhizal fungi (AMF) in the phylum Glomeromycotina are ubiquitous soil microorganisms and obligate root symbionts inhabiting almost all terrestrial ecosystems. The AMF establish symbiotic associations with approximately 80% of vascular plants and with approximately 90% of agricultural plants (Smith and Read, 2008). In the mutualistic association, the fungal partner receives up to 20% of total photosynthates (Allen et al., 2003) and lipids (Bravo et al., 2017) from the host, whereas the plant increases mineral nutrient and water uptake through mycorrhizal hyphae networks (Smith and Read, 2008). Arbuscular mycorrhizal fungi account for 5% to 36% of total soil biomass and 9% to 55% of soil microbial biomass (Olson et al., 1999), and 1 gram of soil contains 10 to 100 m of mycorrhizal hyphae (Gilbert and Johnson, 2017). Mycorrhizal fungal symbionts are important in environmental ecology and agricultural ecosystems, because AMF are ubiquitous and are involved in nutrient cycles (Azcon-Aguilar and Miguel-Barea, 2015).

Establishing arbuscular mycorrhizae (AM) involves a sequence of genetically controlled phases that commences with pre-symbiotic molecular crosstalk resulting in reciprocal perception, followed by host root cells proceeding with considerable functional and structural alterations to accommodate the fungi (Choi et al., 2018). Level of nutrient availability is determinant in establishment and development of mycorrhizal symbiosis. In the mutualistic association, fungi and plants perceive one another *via* interacting molecular signals (Bonfante and Genre, 2015). Before physical contact, strigolactones released from host roots in response to inorganic phosphorus starvation induce AM spore germination, hyphae production, and branching to physically contact roots (Ho-Plágaro and García-Garrido, 2022). Moreover, flavonoids, 2-hydroxy fatty acids, polyamines, and cutin monomers are among the active plant compounds influencing hyphal elongation or branching (Becard et al., 1992; Ghachtouli et al., 1995; Akiyama et al., 2005; Nagahashi and Douds, 2011; Wang et al., 2012; Gutjahr and Parniske, 2013). The AM hyphopodium penetrates into roots and forms arbuscles where nutrients and photosynthates are exchanged (Nadal and Paszkowski, 2013). The AMF develop extensive extraradical hyphal networks in soil, which affect other organisms and root physiology as well as pattern of root exudation.

Although important advances in understanding the molecular regulation of AM symbiosis have been achieved (Ho-Plágaro and García-Garrido, 2022), there is little information on VOCs during mycorrhization. Sun et al. (2015) showed that germinating spores of the AMF *Gigaspora margarita* emit unidentified volatiles, which increase density and number of lateral roots in *A. thaliana* (non-host plant for AMF) and *Lotus japonicus* (**Table 2**). Fungi also modulate host root orientation by releasing sporal VOCs that alter the branch angle of lateral roots, thereby increasing the chances of AM hyphae contacting roots in the rhizosphere (Sun et al., 2015). Because auxins regulate the branch angle of lateral roots, VOCs can trigger the auxin signaling pathway in plants (Roychoudhry et al., 2013). Expression profiles of genes associated with AM establishment and lateral root formation in *L. japonicus* indicate that the gene *LjCCD7*, an important component of the strigolactone synthesis pathway, is stimulated by fungal VOC signals (Sun et al., 2015). Mycorrhizal VOCs increase strigolactone biosynthesis and root proliferation, and secretion of such VOCs in the rhizosphere facilitates AM hyphal identification of roots and increases root colonization. Plant hormones and root volatiles and exudates are important factors modulating interactions between host plants and AMF. Exogenous abscisic acid (ABA) application to mother spores of *Rhizophagus irregularis* substantially increases daughter spore production, and hairy root volatiles considerably increase pre-symbiotic sporulation (Liu et al., 2019). Plants can distinguish between symbiotic and pathogenic interactions in the early stages of colonization and respond by releasing different root VOCs depending on whether the colonization is beneficial or pathogenic (Dreher et al., 2019). Indeed, 93 VOCs exhibit differential responses in *Medicago truncatula* roots treated by the root pathogen *Aphanomyces euteiches* or the mycorrhizal fungus *R. irregularis*. Several VOCs are released specifically in response to *R. irregularis*, such as limonene, which could be a result of the action of specific receptors on plasma membranes (Dreher et al., 2019). Therefore, specific receptors for AMF activate the common symbiotic signaling pathway for symbiotic interactions (Parniske, 2008).

Ectomycorrhizal (EM) symbiosis is another very common mycorrhiza–plant interaction. Many ectomycorrhizal ascomycetes and basidiomycetes form symbioses with approximately 6000 tree species, including beeches, dipterocarps, eucalypts, oaks, pines, and poplars (Brundrett, 2002; Van Der Heijden et al., 2015). In contrast to arbuscular mycorrhizae, ectomycorrhizae support hosts by generating hyphal networks (known as the Hartig net) that surround epidermal cells of emerging lateral roots (Smith and Read, 2010; Tedersoo et al., 2010). A complex signaling dialogue between host plant and fungus is necessary to establish EM. Menotta et al. (2004) showed that 29 volatile compounds, including alcohols, aldehydes, and ketones, are produced during the interaction between the host plant *Tilia americana* and *Tuber borchii* at the pre-symbiotic stage of EM establishment. Terpenoids such as 1-pentanol, 2,3-dimethyldecane, and *p*-isopropylbenzaldehyde—which are the most diffusible compounds in soil (Hiltpold and Turlings, 2008)—are involved in the interaction and therefore may be good candidates for belowground signaling during plant–EM interactions. Indeed, Ditengou et al. (2015) found that those volatiles have roles in the pre-symbiotic communication between roots of a *Populus* host and the fungus *Laccaria bicolor*. The sequiterpene thujopsene generated by the fungus increases *Populus* lateral root formation and root hair length in the pre-symbiotic phase and thus facilitates EM establishment. In addition, thujopsene induces the formation of superoxide anion radicals in the meristematic zone of root tips, whereas the prohibition of fungal sesquiterpene synthesis by lovastatin decreases lateral root formation. Recently, the EM fungus *Tricholoma vaccinum* was found to release geosmin (Abdulsalam et al., 2021), which improves sporulation (Bentley and Meganathan, 1981) and spore germination in AMF (Carpenter-Boggs et al., 1995). Therefore, geosmin may be also important in EM formation.

### Mycorrhizae shape the rhizosphere microbiome

The extensive hyphal network that develops during mycorrhizal colonization alters root morphology and architecture (Schellenbaum et al., 1991; Norman et al., 1996) and increases soil biological activity by what is called the “mycorrhizosphere effect” (Linderman, 1988). Thus, the mycorrhizosphere effect affects soil microbial communities. Mycorrhization causes changes in components of root exudates and therefore shapes soil microbial communities (Badri and Vivanco, 2009). The antifungal activities of mycorrhizal root exudates also promote disease resistance. Zhang et al. (2012) showed that *Glomus versiforme* changes the exudation pattern of cotton roots and contributes to bioactive effects on *Verticillium dahliae* conidial germination. Similarly, direct antibiotic activity of exudates originating from tomato roots colonized by AMF toward *F. oxysporum* f. sp. *lycopersici* has been observed, with nonvolatile citrate and chlorogenic acid as the antifungal substances (Hage-Ahmed et al., 2013). Zhang et al. (2018) demonstrated that *R. irregularis* symbionts emit undefined volatile compounds that directly suppress growth and extension of fungal pathogens such as *F. oxysporum*, *F. graminearum*, *V. dahliae*, and *Rhizoctonia solani.* Furthermore, *Tricholoma vaccinum* (EM fungus) produces VOCs that include the monoterpene limonene and the sesquiterpene β-barbatene, which have antimicrobial properties (Abdulsalam et al., 2021). Notably, plants in natural communities participate in shared or common mycorrhizal networks that enlarge areas accessed by root systems and allow linkages with other plants (Johnson and Gilbert, 2015). In forests, trillions of mycorrhizal rootlets from various forest trees are interconnected by hyphae of different EM fungal species to form extraradicular mycorrhizal networks or wood-wide webs (Selosse et al., 2006; Klein et al., 2016). Molecules likely transported by mycorrhizal networks include small RNAs, hormones or hormone metabolites, ions, peptides, allelochemicals, and particularly defense signals that prime plant resistance to pathogens and herbivores (Johnson and Gilbert, 2015; Hettenhausen et al., 2017; Song et al., 2019; Alaux et al., 2020).

Remarkably, mycorrhizal symbiosis changes plant hormonal homeostasis (Ho-Plágaro and García-Garrido, 2022), because phytohormones are involved in transient plant defense responses essential for homeostatic establishment between AMF and host. Plant hormones can also regulate VOC production in plants. Ethylene, influenced by mycorrhizal colonization (Ludwig-Müller, 2010), not only functions as a phytohormone to modulate volatile biosynthesis but is also emitted into the rhizosphere as a VOC (Chen et al., 2020). Ethylene released from roots or in soil treatments increases the diversity of soil microbes by increasing the number of keystone taxa, such as *Pseudolabrys* spp. (*Alphaproteobacteria*), *Dokdonella* spp. (*Gammaproteobacteria*), and *Catenulispora* spp. (*Actinobacteria*), which leads to changes in soil microbiomes because of changes in production of antibiotics or microbial growth stimulators (Chen et al., 2020). Ethylene also modulates VOC biosynthesis and emissions in plants. For example, ethylene inhibits VOC biosynthesis in potato (Dawood et al., 2014) and rice (Mujiono et al., 2020) subjected to flooding stress. Activation of the salicylic acid (SA) signaling pathway modifies root exudate profiles, which affects soil microbiomes (Martínez-Medina et al., 2017a; Jacoby et al., 2020). Arbuscular mycorrhizal colonization can decrease SA contents in plants, whereas the colonization rate can be inhibited by constitutive SA biosynthesis (Medina et al., 2003). Plant roots can emit large quantities of SA. The SA is dispersed over several centimeters and is transformed into its volatile derivatives, which then alter the structure of microbial communities (Dehimeche et al., 2021; Kong et al., 2021). Notably, Pons et al. (2020) found that phytohormones including cytokinin (isopentenyl adenosine), an auxin (indole-acetic acid), gibberellin (gibberellin A4), and ethylene are also produced by the AM fungus *Rhizophagus irregularis*. Similarly, the EM fungus *Tricholoma vaccinum* emits ethylene and excretes ABA, SA, jasmonates, and indole-3-acetid acid (Abdulsalam et al., 2021). Thus, root VOC emissions are affected by the mycorrhizosphere effect and mycorrhiza-induced changes in phytohormone homeostasis during colonization from the first stage to later stages. In addition, during colonization, mycorrhizal volatiles are released with broad-spectrum and long-term fungistatic efficacy (Zhang et al., 2018). Overall, common mycorrhizal networks (AMF) and wood-wide webs (EM fungi) shape microbiomes in the mycorrhizosphere.

### Mycorrhiza-induced plant volatiles against abiotic and biotic stresses

Arsenic (As) is a prevalent toxic element in natural surroundings and is also used in various industries. The enormous anthropogenic discharge has led to As accumulation in the environment, particularly in waters and soils, seriously threatening crop cultivation and human health in recent decades (Zhao and Wang, 2020). Notably, Li et al. (2021) identified an AM association associated with As volatilization. In *in vitro* cultivation with intact *Medicago sativa* plants colonized by *R. irregularis*, the AM symbiosis methylates and volatilizes inorganic As to a variety of organic forms, including dimethylarsinic acid, dimethylarsine, and trimethylarsine, modulated by *RiMT-11* (a gene of *R. irregularis* encoding the methyltransferase type 11 protein). As a result, the AM symbiosis increased host plant tolerance to As stress. In the process of detoxification, methylation can produce trivalent methylarsenites as intermediate products, which are even more toxic than inorganic As (Li et al., 2021). The methyltransferase evolved to generate extremely toxic trivalent As species that acted as antibiotics to destroy competitors in the primitive anaerobic earth (Chen et al., 2017). As atmospheric oxygen levels increased, the trivalent forms were oxidized to pentavalent methylarsenicals, which converted methylation into a process of detoxification. Because AMF are very ancient symbiotic fungi, whether As methylation capability originally evolved to benefit both plants and AMF by eliminating pathogenic microbes remains a topic of great interest.

Mycorrhizae affect concentrations and composition of root VOCs in various plant species such as *Sorghum bicolor* (Sun and Tang, 2013), *Medicago truncatula* (Dreher et al., 2019), and *Vitis vinifera* (Velásquez et al., 2020a). In addition, mycorrhizal effects on VOC production differ depending on the AMF species (Sun and Tang, 2013; Volpe et al., 2018) and the plant species (Meier and Hunter, 2019). The mycorrhizal fungus genus *Glomus* may increase biotic stress tolerance in various plants attacked by herbivores (Dowarah et al., 2021).

Methyl salicylate synthesized from SA modulates plant defenses to environmental stresses and disease resistance by stimulating defensive compound production or by activating SA-signaling defense (Raskin, 1992). Mycorrhiza-induced increases in methyl salicylate may be important, because it is a volatile that is an elicitor linked with induced resistance to plant diseases and has been used in plant protection (Tang et al., 2015; Kalaivani et al., 2016). Methyl salicylate can control some aphid species and prevents attacks on host plants (Sasso et al., 2007; Babikova et al., 2014). Under drought stress and aphid infestation, increases in methyl salicylate levels in mycorrhizal plants led to greater attraction of the aphid parasitoid *Aphidius ervi* than that in nonmycorrhizal ones (Volpe et al., 2018).

In addition to methyl salicylate, mycorrhizal colonization increases other volatiles associated with plant defenses against pathogen/herbivore attack or drought stress, including benzaldehyde, geraniol, (E)–2–hexenal, and 3–hexenal in grapevine plants (Velásquez et al., 2020b). Application of C6–volatiles, such as (E)– 2–hexenal, leads to increased resistance against the necrotrophic fungus *B. cinerea* (gray mold) in *Arabidopsis* by suppressing fungal development and triggering the plant defense response (Shiojiri et al., 2006). Benzaldehyde is a VOC with nematicidal and antifungal properties (Shaukat et al., 2005), and geraniol is associated with plant defense, triggering apoptosis–like cell death, with fragmentation of nuclei and DNA (Izumi et al., 1999). Geraniol may be in high concentrations in various plant organs because it is a precursor for diverse monoterpenes (D’Onofrio et al., 2016), whereas benzenic compounds are the major components of some essential oils and are linked to plant defense and reproduction (Dudareva et al., 2004; Carvajal et al., 2016).

D-Limonene, p-xylene, and 1,3-diethylbenzene increase in *Elymus nutans* plants colonized with AMF (Zhang et al., 2022). Under insect attack, 1,3-diethylbenzene and D-limonene can be generated to strengthen plant defenses and repel insects (Agut et al., 2015; Kigathi et al., 2019; Mitra et al., 2021). The compound p-xylene is primarily an attractant to natural enemies of herbivorous insects (Li et al., 2022). Levels of monoterpenes increase substantially in *Funneliformis mosseae-*colonized roots of grapevine (Velásquez et al., 2020a). Terpenoids are important in above- and belowground tritrophic interactions, because terpenoids attract parasitoids and predators of herbivorous insects (Palma et al., 2012; Penuelas et al., 2014). Notably, there are increases in monoterpene alcohols associated with plant defense, such as p-mentha-1.8-dien-7-ol, myrtenol, and p-cymen-7-ol. Monoterpenes of p-menthane are widely dispersed in plants and are major components of various essential oils and plant extracts with biological activity in plant defense (Lange, 2015). Terpenoids are also associated with function and formation of mycorrhizal symbiosis. Terpenoid accumulation is observed in AM roots of many plants, particularly in root cortical cells with collapsed arbuscules (Akiyama and Hayashi, 2002; Fester et al., 2002). In addition, mycorrhization causes a sharp increase in transcripts of two early enzymes (DXS and DXR) of the 2-methyl-D-erythritol-4-phosphate pathway in host roots (Walter et al., 2000; Hans et al., 2004). Nevertheless, mechanisms associated with fluxes of volatile terpenoids with different roles in mycorrhizal symbiosis remain unknown (Kapoor et al., 2017).

## Methodology on belowground research of volatile organic compounds

Volatile organic compounds are typically released as a blend of compounds that is diluted in the environment around a plant. In recent years, evidence increasingly shows that belowground plant VOCs have important roles in trophic interactions. Although advances have been made in techniques to sample aboveground VOCs (Raguso and Pellmyr, 1998; Tholl et al., 2006; Penuelas et al., 2014; Materic et al., 2015), sampling belowground volatiles is more difficult because of the nonhomogeneous trapping environment (van Dam et al., 2016; Tholl et al., 2021; Sharifi et al., 2022).

Understanding the roles of belowground VOCs in belowground communication networks is attracting increased attention (van Doan et al., 2021). It is necessary to invest in advanced methodology and instrumentation to capture and fully analyze belowground VOCs (Sharifi et al., 2022). Currently, most research on root VOCs uses ground root material with the caveat that the total profile of volatiles in root tissue was analyzed rather than emissions of volatiles (Gfeller et al., 2019). However, the approach can detect chemicals that may be not induced by major root damage (Tholl et al., 2021). In this section, we describe the most challenges in sampling belowground volatiles and the recent progress in sampling and analyzing belowground VOCs; we further describe a promising experimental design for future research on belowground VOCs.

Compared with volatiles in relatively homogeneous aboveground environments, belowground VOCs are a mixture of volatiles from plant roots, bacteria, fungi, parasites, herbivores, and predators (Delory et al., 2016a; van Dam et al., 2016). Therefore, it is difficult to evaluate the exact effects of specific original VOCs on trophic interactions. It may be necessary to distinguish among origins of VOCs when the goal is to assess effects of fungal VOCs on plant–root–insect interactions. Effects of fungal VOCs may be synergistic or antagonistic, depending on whether VOCs originate from roots, insects, or other soilborne organisms. In addition, belowground VOCs are not only diluted by the surrounding environment (Turlings et al., 2012; Tang et al., 2019; Ehlers et al., 2020; Erktan et al., 2020; Wester-Larsen et al., 2020) but also depend on microbes such as those producing and consuming VOCs (Raza et al., 2016; Bier et al., 2017; Schenkel et al., 2018; Abis et al., 2020; Gutiérrez-Santa et al., 2020). Therefore, a sampling method suitable to collect targeted belowground VOCs is required that can overcome the challenges of a nonhomogeneous environment (van Dam et al., 2016; Tholl et al., 2021).

The method used to sample belowground VOCs strongly depends on the research purpose. However, there are two main approaches to analyze root volatiles. Volatile organic compounds are measured either directly in the soil matrix or after root excavation, extraction, and analysis under lab conditions. In this review, the aim was to explain the most applicable methods to directly collect belowground volatiles in the soil matrix (Gfeller et al., 2019; van Doan et al., 2021; Tholl et al., 2021), and describe a detailed comparison between the approaches (**Table 3**). There are two approaches to measure VOCs, with one method a dynamic “push–pull” system and the other a passive method using solid-phase microextraction (SPME) (Tholl et al., 2021).

**Table 3:** Advantages and disadvantages of dynamic and passive methods to collect volatile organic compounds (VOCs) in belowground environments.

Method to collect belowground VOCs	Advantages	Disadvantages	After sampling/pre-analysis process
Dynamic sampling (Tholl et al., 2021) Gas chromatography–mass spectrometry (GC-MS), Pull/push–pull systems (Adsorbent traps, Trapping Super-Q)	➢Separate sampling and analysis times ➢Controlled collection and pre-concentration of VOCs ➢Quantitative and qualitative analyses ➢Repeatable sample analysis ➢Application of miniature devices (e.g., Super-Q trap)	➢High Cost ➢More challenging to apply in the field or other places ➢Sampling requires equipment (pumps, flow meters, charcoal filters, VOC traps) ➢Use of organic solvents in solvent elution and liquid injection	➢Method collects volatile mixtures, need to future step to distinguish original VOCs ➢Trap>>elute traps with solvents for liquid injection or use thermal desorption of traps>>GC-MS or Gas Chromatography–Time-of-Flight Mass Spectrometry (GCxGC-Tof MS) analysis
Passive sampling (Tholl et al., 2021) GC-MS, SPME, Polytetrafluoroethylene (PTFE) tubing	➢Low cost ➢Miniature sampling devices, sensitive, cost effective ➢No consumption of organic solvents, clear spectrum of VOCs without solvent background interference ➢Sampling is a snapshot of the VOC current state rather than for a time interval	➢Separate sampling and analysis times ➢One-time only sample analysis due to thermal desorption (SPME) ➢Limited quantitative analysis ➢Adsorbent preference for analytes	➢Method collects volatile mixtures>> directly measure with thermal desorption of fibers or tubing>>GC-MS or GCxGC-Tof MS analysis

**Figure 2:**
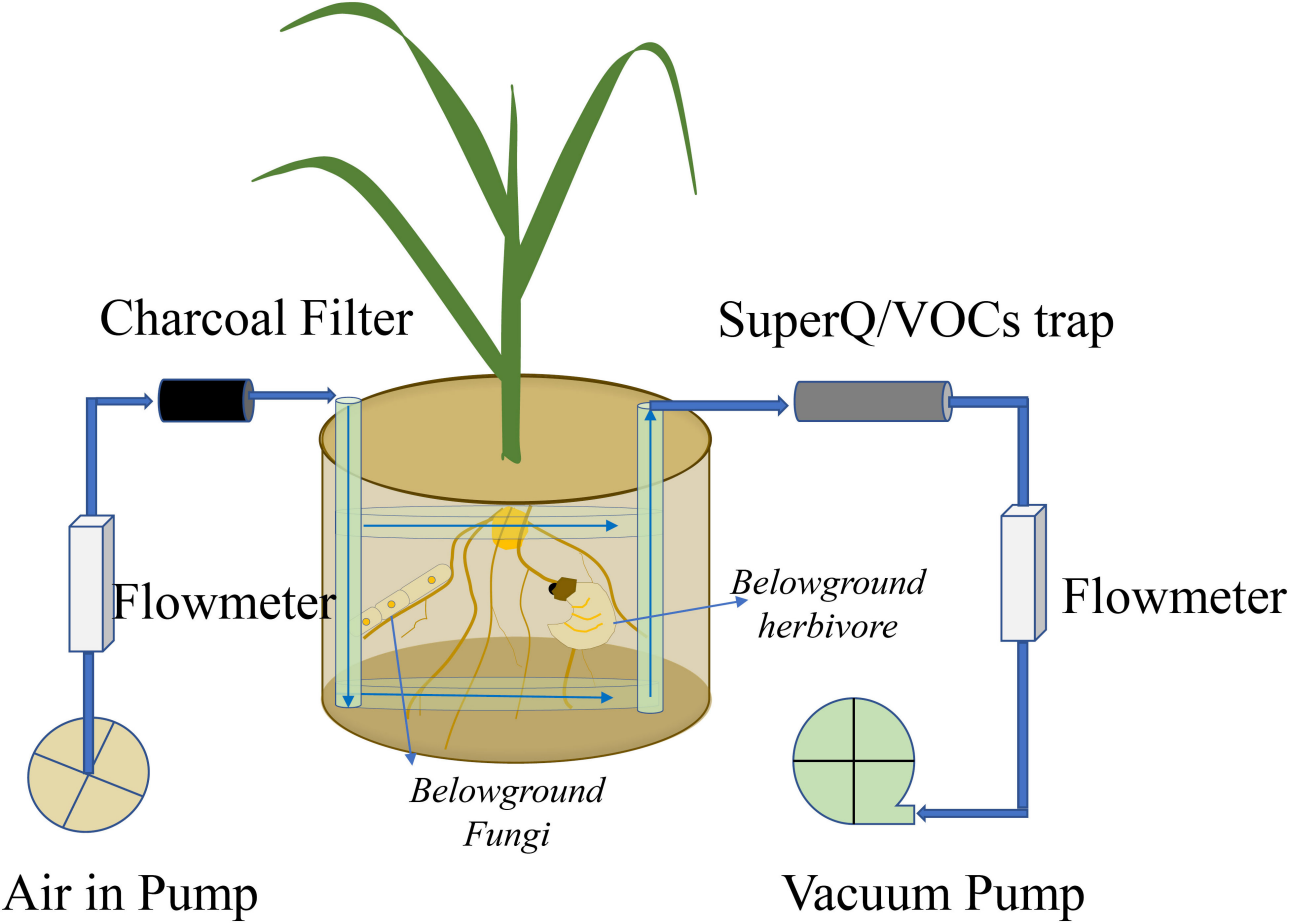
Volatile organic compound (VOC) emissions from roots, soilborne organisms, and soil matrix are collected by a push–pull system. The VOCs are trapped by a Super-Q trap.

The technique using a “push–pull” system collects all belowground VOCs, including those emitted from roots, soilborne organisms, and soil matrix), by using clean-air flow through the belowground system, with VOCs trapped by a Super-Q filter (**Figure 2**) (Hiltpold et al., 2011; van Doan et al., 2021). Briefly, spherical pots are connected to multiple air delivery systems, and volatiles are trapped on Super-Q filters (25 mg of Super-Q adsorbent, 80–100 mesh; Alltech Assoc., Deerfield, IL, USA). Cleaned humidified air is pushed through the system at a rate of 1 L min^-1^ and pulled through Super-Q traps at a rate of 0.7 L min^-1^. After collection, Super-Q filters are rinsed with 150 μL of dichloromethane. N-octane and nonyl-acetate (Sigma, Buchs, Switzerland) are added as internal standards (200 ng in 10 μL of dichloromethane). Root volatiles are analyzed by a gas chromatograph coupled to a mass spectrometer (GC-MS, Agilent 7820A GC coupled to an Agilent 5977E MS; Agilent Technologies, Santa Clara, CA, USA). Putative volatile identification is obtained by comparing mass spectra with those of the NIST05 Mass Spectra Library (van Doan et al., 2021). Other sorbent materials are also frequently used in dynamic approaches to trap VOCs and are summarized by Tholl et al. (2006); however, Tenax TA and Carbopack B are used even in passive methods to sample belowground VOCs (Martín-Sánchez et al., 2020).

**Figure 3:**
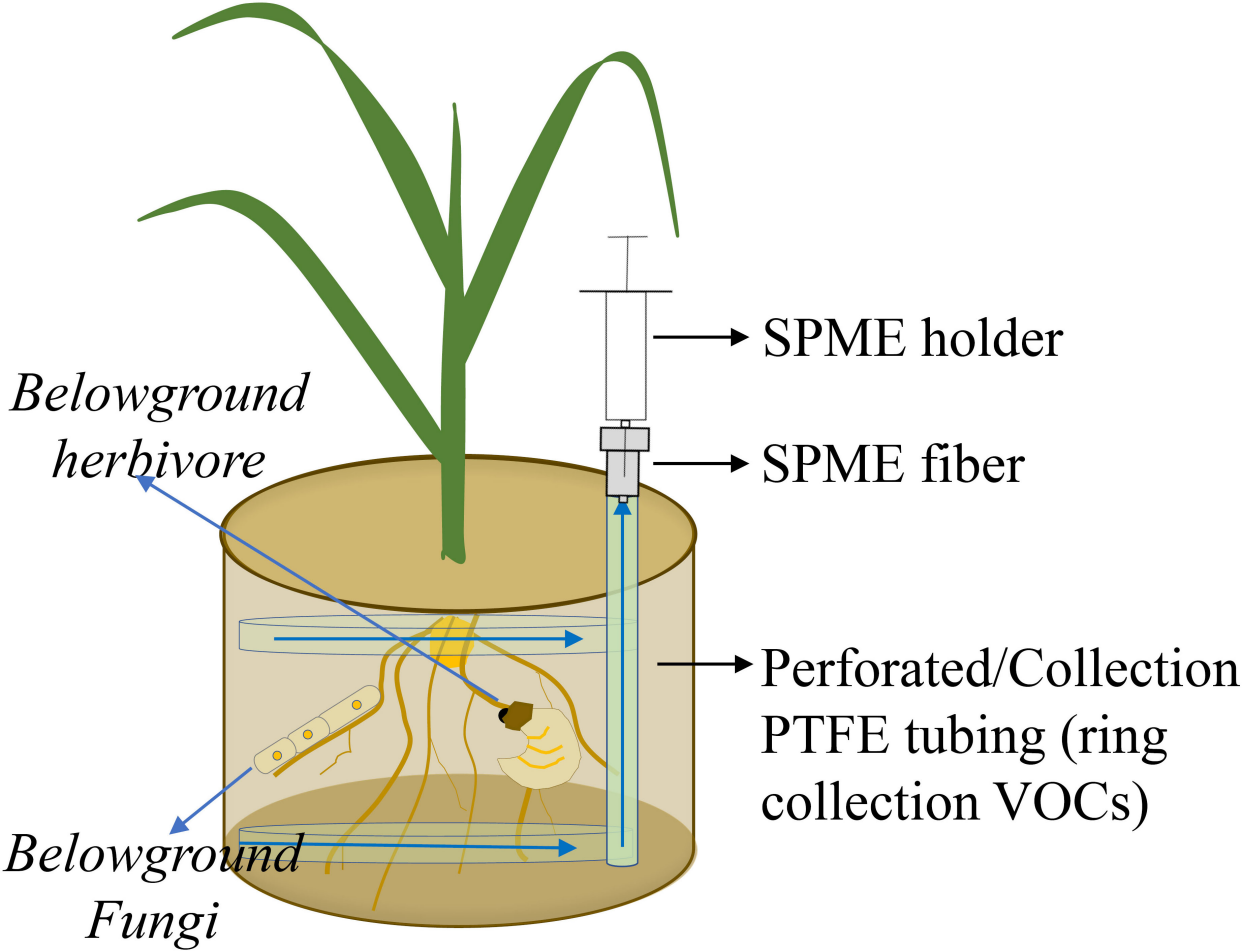
Volatile organic compound (VOC) emissions from roots, soilborne organisms, and soil matrix are collected by a passive system. VOCs are trapped by a solid phase micro extraction (SPME) fiber. Polytetrafluoroethylene (PTFE).

Of possible passive sampling methods, a less complex system is one in which an SPME fiber (coated with 100-μm polydimethylsiloxane; Supelco, Bellefonte, PA, USA) is inserted into a gap of a pot and exposed to belowground VOCs for 60 min at room temperature and then transferred to another pot for 60 min to collect VOCs (**Figure 3**). An incubated fiber is immediately analyzed by GC-MS using an Agilent 7820A GC interfaced with an Agilent 5977E MSD. Volatile organic compounds are tentatively identified by comparing mass spectra to library entries of the National Institute of Standards and Technology (NIST 14) and an external standard library (Gfeller et al., 2019). The advantage of such a system is that all emerging VOCs from the belowground mixture are collected, and thus, most VOCs are generated by targeted organisms. However, using SPME is at best a semi-quantitative approach, and depending on VOC composition, different SPME fibers should be tested because of differences in fiber affinity for classes of VOC compounds. In addition, extraction times and temperatures are important and need to be optimized, because high temperatures and long extraction times cause desorption of VOCs that have relatively low fiber affinity or low boiling point from an SPME fiber during extraction.

To understand the mechanisms of belowground VOCs in ecosystems, we need to measure the belowground VOCs directly measured in a certain time. Danner et al. (2012; 2015) directly measured VOCs released from root herbivore damage in cuvettes on the top of the soil at the stem and root interface. Acton et al. (2018) measured VOCs by using airflow generated in a root glass chamber filled with a potting substrate. All belowground VOCs emitted to the environment in a certain time can be measured by proton transfer reaction mass spectrometry (PTR-MS) (Majchrzak et al., 2018; Tholl et al., 2021; Sharifi et al., 2022).

The PTR-MS method also has disadvantages, because it characterizes only mass-to-charge ratio (m/z) of VOCs and not the exact molecular identity. In addition, one molecular formula may represent different structures, which cannot be discriminated by PTR-MS. Some small-chain alkanes are also not detected by the technique. Therefore, the PTR-MS method is generally used simultaneously with GC-MS to determine chemical identities of volatiles from the m/z data (Sharifi et al., 2022).

**Figure 4:**
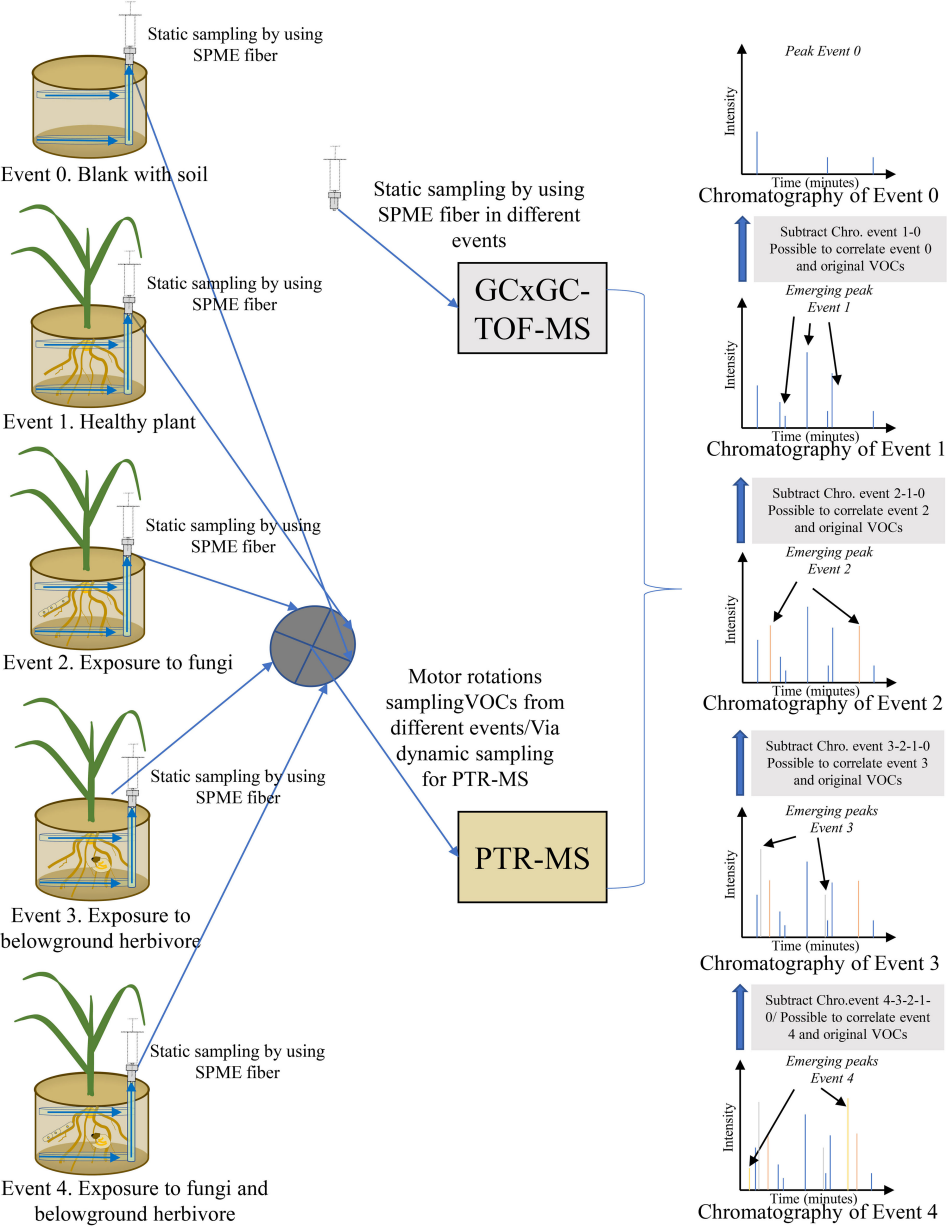
Schematic illustration of an *in-situ* design to collect and analyze belowground volatile organic compounds (VOCs) by a combined technique. Original VOCs are distinguished by subtracting chromatographic peaks of certain events.

The challenge with passive and dynamic methods is in collecting the many different original belowground VOCs and establishing emission origins. To meet the challenge, a new experimental setup and methods can be optimized to minimize the disadvantages of the two approaches (**Figure 4**). An experimental system can be set up in which both sampling approaches are used simultaneously, and different treatments or events (blank pot with soil only, healthy plant, plant exposed to fungi, plant exposed to belowground herbivore, plant exposed to fungi and belowground herbivore) are used to compare differences in VOCs. After comparison and subtraction of VOC patterns of different events, emission origins and abundance of VOCs can be established. Sharifi et al. (2022) presented an *in-situ* design suitable for sampling belowground VOCs that used a perforated polytetrafluoroethylene (PTFE) tube exposed to communities of plant roots and soil microorganisms. The tube is placed in a pot before sowing seeds to avoid disturbing the soil and rhizosphere when belowground VOCs are collected. Belowground VOCs are collected by an SPME syringe extracted *via* a network of tubing. To separate the original VOCs, an experimental setup is suggested according to Sharifi et al. (2022) that can continuously sample different treatments or events by SPME fibers inserted into PTFE tube systems. After collection of VOCs, SPME fibers are analyzed in a cyclic manner by GC-MS or GCxGC-TOF-MS. The tube systems can also be sampled by a dynamic approach in which different treatments are connected by motor rotation switch valves to a PTR-MS. With this approach, SPME collections obtained by frequent static sampling cycles can provide a good approximation of real-time resolution in emissions of individual VOCs, in addition to VOC composition and abundance. The PTR-MS can characterize actual real-time continual emissions of different events. After subtraction and comparison of different events, VOC origins and emissions can be characterized on the basis of the combined sampling and analysis methods to yield a highly accurate approximation of VOC patterns and emission origins (**Figure 4**).

## Conclusion and future perspectives

Volatile organic compounds emitted by plant roots and pathogenic and beneficial fungi, particularly mycorrhizal fungi, can shape trophic interactions in belowground systems. Fungal VOCs mediate plant growth, metabolites, and consequences of interactions between insects, pathogens, and plants. In this review, an approach using combined methods is proposed to collect VOCs and analyze the effect of each originated VOC in real-time. With the approach, the effect of each originated VOC on belowground trophic interactions can be precisely evaluated. Because of the essential roles of VOCs in inter- and intraspecific communication, using VOCs of certain fungal species may be a promising and sustainable way to reduce the incidence of diseases derived from soilborne phytopathogens. In addition, using fungal VOCs to increase plant tolerance against abiotic stresses is an area for future research with great potential. Despite various reports on interactions between belowground VOCs derived from fungi and plants and root VOCs and fungi that result in benefits for one or both partners, the actual mechanisms involved remain unknown. Therefore, the molecular mechanisms responsible for volatile production by VOC producers (plants and fungi, including fungal symbionts), perception by VOC receivers, and genetic reprogramming of VOC receivers need to be investigated further. Moreover, there are few reports on VOCs during mycorrhization, which should be a research area with great potential interest because of the importance of AMF in agriculture and ecosystems. In addition, most knowledge on VOC emissions by fungi is based on single strains under laboratory conditions, which can differ from rhizospheric conditions with complex microbial communities. Therefore, to facilitate practical VOC application, inoculated strains should be integrated into complex rhizosphere communities in order to mimic the natural conditions in soil.

## Author contributions

ND: conceptualization, outline, writing abstract, mycorrhiza section, and conclusion, edit, and revisions. HV: conceptualization, outline, writing VOC pathogen section, and format. CD: conceptualization, outline, and writing introduction and method section. KH: writing methods and revisions. KL: conceptualization, outline, and revisions. KP: conceptualization, outline, revisions, edit, and corresponding author. All authors contributed to the article and approved the submitted version.
